# Supplementary material for: A vaccine using Anaplasma marginale subdominant type IV secretion system recombinant proteins was not protective against a virulent challenge
Source: PLoS One. 2020 Feb 21;15(2):e0229301. doi: 10.1371/journal.pone.0229301 (PMC7034839; doi:10.1371/journal.pone.0229301)
Supplement: S1 Raw images — (PDF) [file pone.0229301.s001.pdf]

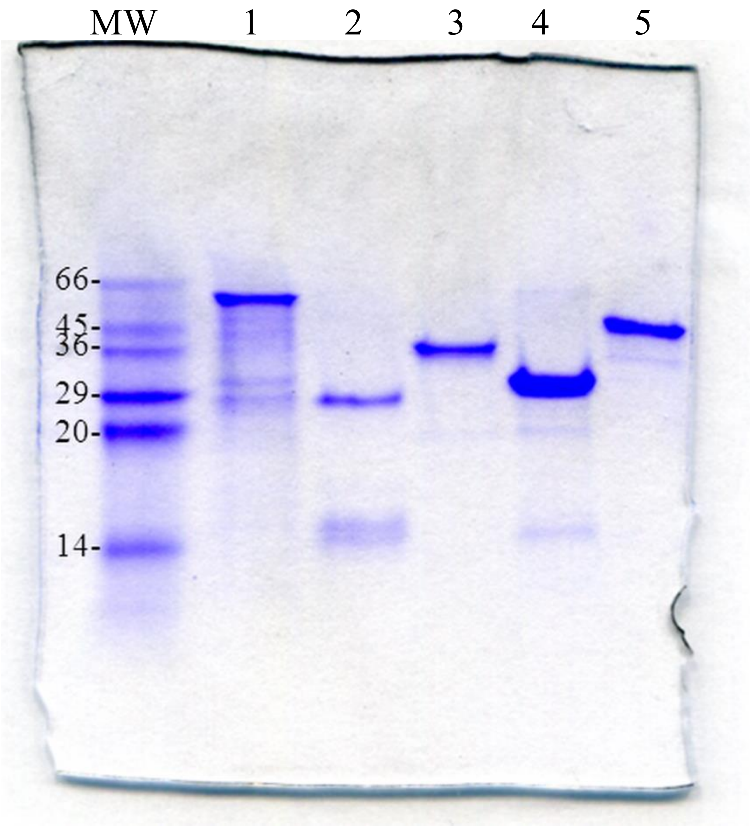

Fig 1A. SDS-PAGE, stained with Coomassie Brilliant Blue R-250. MW: molecular weight marker (kDa); lane 1: VirB10; lane 2: tVirB9.1; lane 3: VirB11; lane 4: tVirB9.2; lane 5: Ef-Tu.

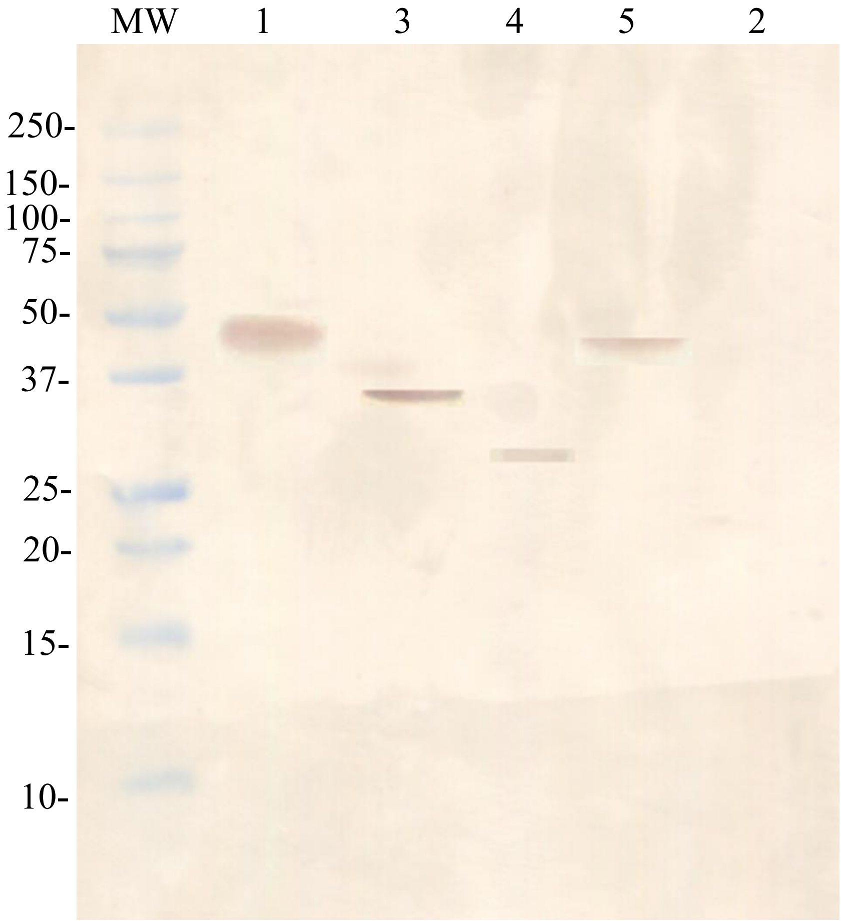

Fig 1B. Western blot revealed with anti-His-tag MoAb. MW: molecular weight marker (kDa); lane 1: VirB10; lane 2: tVirB9.1; lane 3: VirB11; lane 4: tVirB9.2; lane 5: Ef-Tu.

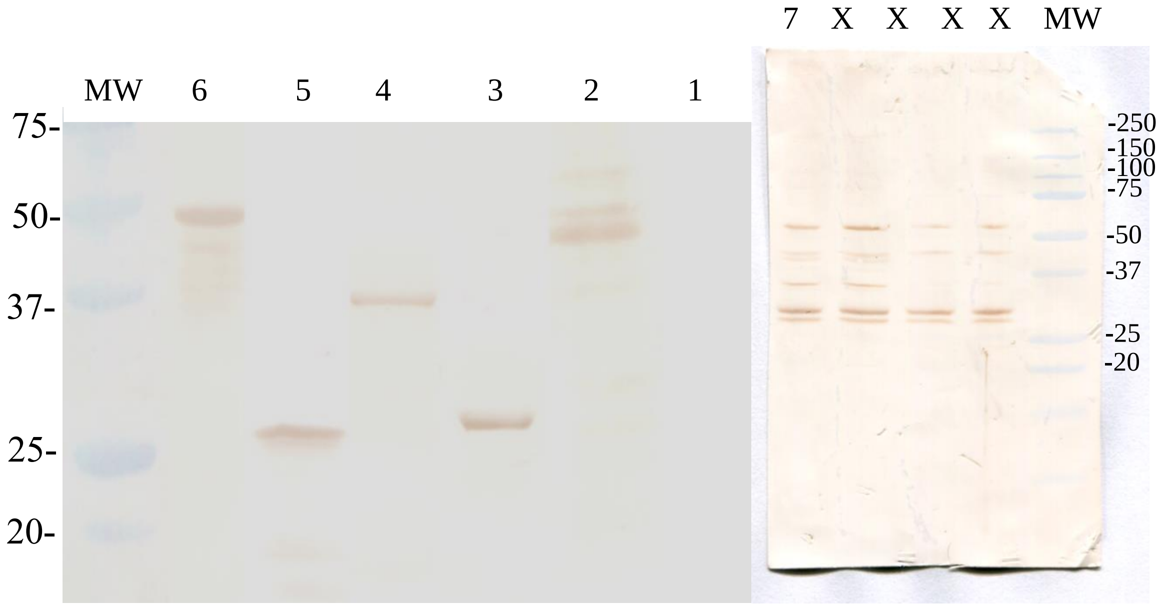

Fig 3. Reactivity of sera obtained 7 days after the fourth inoculation from cattle inoculated with recombinant proteins/Quil A (Group 1) or recombinant proteins/Montanide (Group 2) by Western blot. A representative steer of group 1 is shown. MW: molecular weight marker (20, 25, 37, 50 kDa). Lane 1: MSP5; lane 2: Ef-Tu; lane 3: tVirB9.2; lane 4: VirB11; lane 5: tVirB9.1; lane 6:VirB10; lane 7: *A. marginale* crude antigen. Sera were diluted 1/100 and the reaction was detected with anti-bovine IgG peroxidase conjugate.
