# Supplementary material for: A vaccine using Anaplasma marginale subdominant type IV secretion system recombinant proteins was not protective against a virulent challenge
Source: PLoS One. 2020 Feb 21;15(2):e0229301. doi: 10.1371/journal.pone.0229301 (PMC7034839; doi:10.1371/journal.pone.0229301)
Supplement: S1 Table — The signal peptide of VirB9.1 and VirB9.2, excluded from tVirB9.1 and tVirB9.2, are highlighted with horizontal gray bars. The transmembrane helices from VirB10 are indicated in italics. (PDF) [file pone.0229301.s002.pdf]

VirB9.1	MKKAFMVCAVALLCSSAAFGKQEPRSIAADDHIKIINFNPQSIHRYTGFYGYQSSILFESGEVI<br>DTVSMGDSTGWQLVPKGNRLFIKPVGDNADTNVTIITNRRVYYFELHAEEASGLDDPRLAYEVR<br>FVYPAASSVDAASSSDLGGGVSFPTYQNDVPDLSDPEVAKKGLNFDYSVSHTAGSANIVPIRVF<br>DDRKFTYMQFSNVNGDLPSIFNVDAEGYESLVNFRIVGDYVVVERVSPAFTLRYGSSTACVFNE<br>KLYRTSSTSRRGRG
VirB9.2	MNFYKNLLACSALLTVVFTGGVAQSAVSGGAPVSVDSRIKTFVYSPNEIFTVVFNHGYHSFIEF<br>SKGETIKVMAMGDSVHWKVKPVDNKLFIMPLEREGKTNMLVETNKGRSYAFDLVSKSAGPDAAG<br>YKEVADELGRVDSPLLDMAYVVRFYYPDNNREFDLKGAGLADLSAPSLAKNPNSGEVTVRPNAT<br>GKNYVYSASSADATIVPVKTFDDGALTYFQFYDNNKVIPKVFSVGRHGKKVPCRMLLLKGYVII<br>EGVHKRLYLDYGKSGVEVVNTVL
VirB10	MSLGMSDETKDNNYGDGVEESVNVVGVH**KSKKLFVVLVVCAITGMAY**YMFFRGSGTTETSEEPQ<br>QVIEKQDVDKLLKESEAPAQETAPRILTPPPKLPDLPPLVMPTAPELPTLARIAKKKKEEPVVE<br>ETKEILPPAAESFFEPELQRRPMEDDGPPQHIPMPYRPGGGAIPEPVPSFLGYDREKRGTPMIV<br>LGGGGDGGPSEDGGGQGTDSRFSTWSTLDGTSSPSVKATRVGDPGYVILQGHMIDAVLETAINS<br>DIPGVLRAIVSRDVYAEAGNMVMIPKGSRLIGSYFFDASGNNTRVTVSWSRVILPHGIDIQINS<br>AGTDELGRNGSAGFIDTK**MGNVLTSTILLAGVSMGTA**FVTSKIPALQSEIKDTTEEKGEKKKEE<br>KSSTLPVKIVSDAVKDFSESMKALIKKYVDTSKPTIYVDQGTVMKVFVNQDIVFPREAVRR
VirB11	MTAGYAALETYLEPLQSIFAEDGVNEISINRECEVWVENRGDIRCERIESLTLSHLKALGRLVA<br>QATEQKLSEETPLLSASLPNGFRVQVVFPPACEGDKVVFSIRKPSTVQLSLDDYEKMGAFSHVA<br>QQGRKAMDVNKQLSELLDSGDIKSFIELAVLSKKNIIVSGGTSTGKTTFTNAALRVIPKDERII<br>TVEDSREIALDHPNRVHLLASKGGQGRAKVSTQDLIEACLRLRPDRIIVGELRGAEAFSFLRAI<br>NTGHPGSISTLHADTPRMAVEQLKLMVMQASLGLPPDQIVSYITNIVDVIIQLKRESGGVRHVA<br>EIMFTKCSQGND
Ef-Tu	MTEGRKPHINVGTIGHVDHGKTTLTAALTTVLTRRLSGANKVVKYDEIDKAPEERARGITISTA<br>HVEYETEGRHYAHVDCPGHADYIKNMITGAAQMDVAILVVSATDGAMPQTREHILLAKQVGVKD<br>IVTWINKCDVVEDEEMLSIVEMEVRELLSNYGYDGDGVDVVRGSAVKALEESSDGPWSEKIMEL<br>VGALEKIELPVREKDKPFLMSVEDVFSIPGRGTVVTGRIERGVIKVGDKVDIVGLRDLQSTVCT<br>GVEMFHKALETGEAGDNAGILLRGIKKEDVERGQVLSAPGQIRSYKAFKAEVYILKKEEGGRHT<br>PFFSNYQPQFYVRTTDVTGSIKLPSGVEMVMPGDNLSIEVALDKPVALDKGLR
