# Supplementary material for: A vaccine using Anaplasma marginale subdominant type IV secretion system recombinant proteins was not protective against a virulent challenge
Source: PLoS One. 2020 Feb 21;15(2):e0229301. doi: 10.1371/journal.pone.0229301 (PMC7034839; doi:10.1371/journal.pone.0229301)
Supplement: Attachment — Submitted filename: PlosOne 19-26977_reviewers.docx [file pone.0229301.s003.docx]

Dear Editor,

Please find below the manuscript "A vaccine using Anaplasma marginale subdominant type IV secretion system recombinant proteins was not protective against a virulent challenge" PONE-D-19-26977, after complete correction.

The authors developed two formulations containing A. marginale type IV secretion system (T4SS) subdominant proteins: VirB9.1, VirB9.2, VirB10, VirB11, and Ef-Tu T4SS beind expressed in  E. coli.

This proteins were absorbed on a Quil‑A® adjuvant (aqueous saponin adjuvant - G1) and and Montanide ISA 201 ready-to-use adjuvants for Water-in-Oil-in-Water emulsion (G2) and control groups Quil ‑ A® (G3) and ISA 201 (G4), respectively.

Recent reports describe that conserved membrane proteins that are subdominant in Anaplasma species, such as VirB9 and VirB10, are constituents of the Type 4 secretion system (T4SS) that is conserved amongst many intracellular bacteria and performs essential functions for invasion and survival in host cells (Crosby et al 2018). Therefore, here this study is important.

In addition, the work used free healthy cattle and also splentomized calves with parasitized erythrocytes from A, marginale, besides to explain the participation of constituents of the Type 4 secretion system (T4SS) on the immune response against A marginale virulent.

However I would like to request some explanations:

line 52 -54 " Such protection was correlated with induction of high titers of IgG2 opsonizing antibodies against A. marginale surface epitopes and macrophage activation mediated by CD4+ T cells [4,11,12]". To compare the works (4,11,12) and the challenge test employed. Thus, it is important to insert on the discussion section.

line 94 - 100 and 241 -267 "In silico analysis of T4SS proteins sequences". I would suggest the authors to consider the inclusion of figure or a table with the sequences.

line 172 - 182 "Immunization...I would like that authors described about the adsorption methods of the vaccine formulations employed.

It´s necessary to describe on the material and methods section.

Line 187 - 188 "Each challenge dose was adjusted to 107 parasitized erythrocytes in a 2 mL final volume and inoculated SC to the cattle, on day 105 after the first inoculation.

Why? Others reports (Santos et al 2013; Crosby et al 2018) employed 1 x 10 5 for both dominant and subdominant epitopes. it is important to insert discussion section.

line 341-349 "Despite the correlation between IgG2 and protective immunity, both vaccine formulations evaluated in this work induced a strong IgG2 response but failed to mitigate the course of infection. Other subdominant proteins from the OM, AM854 and AM936, were able to induce an immune response comparable to that induced by the whole OM, based on high levels of IgG2; however, they also failed to protect against the challenge [37]" Here, I would like that authors described about the IgG2 production by dominant epitopes, and to compare with obtained data this work (subdominant epitopes).

line 377 - 383 "Another cause of vaccine failure in this study may have been the inability of the immunogen to generate antibodies to block the parasite entry to the host cell. The most successful vaccines target highly conserved epitopes required by the pathogenic parasites for their host cell entry [42]". Here, I would like that authors described about the MSP-2 variants and the CD4(+)-T-cell epitopes that may be responsible for all or part of the A. centrale vaccine efficacy.
