# Supplementary material for: A vaccine using Anaplasma marginale subdominant type IV secretion system recombinant proteins was not protective against a virulent challenge
Source: PLoS One. 2020 Feb 21;15(2):e0229301. doi: 10.1371/journal.pone.0229301 (PMC7034839; doi:10.1371/journal.pone.0229301)
Supplement: Attachment — Submitted filename: Response to Reviewers.docx [file pone.0229301.s004.docx]

# Reviewer #1: Dear Authors,

# This clearly written manuscript presents negative data regarding an immunization and challenge using primarily the type 4 secretion system components of *Anaplasma marginale*. Overall this is nicely done work, though a few things require strengthening or clarification.

# R. The authors need to more clearly demonstrate that they are measuring the immune response directed against the recombinant proteins and not the His tag following immunization. Though it is mentioned on line 227 that the sera were adsorbed with *E. coli* lysate, it is not clear that the expressed His tag was included in the *E. coli* lysate. Additionally, there are no controls to demonstrate that the adsorption was effective in removing the anti-his tag antibodies from the immune serum. This must be clearly addressed in the indirect ELISAs measuring total IgG, IgG1 and IgG2 and the western blot (Fig. 3) in the manuscript.

**A.** We made changes in the manuscript. Briefly, in the study we used three recombinant proteins (SAG1, MSA2c and MSP5) for the adsorption of antibodies anti-His-tag and for the control of the adsorption step. These proteins were expressed in *E. coli* with His-tag and were purified with Ni^2+^-NTA agarose. The sera were adsorbed with an *E. coli* lysate expressing SAG1 protein of *Neospora caninum*. Moreover, we added MSP5 of *A. marginale* as control to determine the presence of anti-His-tag antibodies in the WB assay (Fig 3) and we used *A. marginale* MSP5 and *Babesia bovis* MSA2c recombinant proteins to control the presence of antibodies anti-His-tag in the iELISA assay (Fig 2). These specifications were detailed in the Material and methods and Results sections.

# R. Ef-Tu is an elongation factor and has been identified as a vaccine candidate for *A. marginale*. However, it is not part of the T4SS, this should be clearly explained in the introduction, abstract and discussion.

**A.** We made the changes.

# R. It would be useful to use *A. marginale* protein from infected RBCs in a western blot to confirm that the antibody response to the recombinant proteins resulted in antibodies that can bind *A. marginale* proteins.

**A.** We added in the WB assay (Fig 3) a control of interaction of the post-immunization antibodies with a crude antigen of *A. maginale* purified from the blood of a splenectomized calf with 80% of parasitemia. The crude antigen was purified following the protocol described for the Card agglutination test.

# R. Line 209 and 219: A serum dilution of 1:10 (total IgG) and 1:20 for IgG1 and IgG2 is a very low dilution and suggests that the antibody response was potentially non-specific and weak. Please address this more fully in the manuscript.

**A.** Although the dilution of the sera is low, we adsorbed the sera before performing ELISA and WB, with an *E. coli* lysate expressing SAG1. Then, we demonstrated the specificity of antibodies by the lack of reaction of sera with MSP5 and MSA2c by ELISA and WB (Fig 2 and Fig 3) and the reaction with proteins obtained from *A. marginale* crude antigen (Fig 3)*.* This point was included in the discussion.

# R. Line 215: Please more fully describe the origin of the negative sera? Is this pre-immune serum from the calves in the study or are these sera from other known negative animals?

**A**. The description of the negative sera was included in the manuscript (line 235)**.** Pre-immune sera from cattle were used in this study.

# R. Line 226 and throughout the manuscript- It is difficult to put the results in context because the timeline is generally referenced based on the number of days following the first inoculation. For example, 28 days following the first inoculation is same as one week after the second inoculation. In this case is important for the reader to know that a second immunization was done and the sera were collected one week later in order to put the results in context. Please edit through the document in order to put the timeline into the context of the results.

**A.** We made the changes in the manuscript.

# R. Line 227 and Fig. 3: What was the serum dilution used for the bovine immune serum in the western blot? Please include a control on the western blot indicating that absorption was sufficient to remove the anti-his tag antibodies. Please clarify why Msp5 is used as the negative control? Does it have a His-tag? Please include the production of Msp5 in the methods. Please include pre-immune serum as a negative control.

**A.** We included the data in the manuscript. The dilution of sera in the WB was 1:100. As control of anti-His-tag antibodies removal, MSP5-His-tag was included. MSP5 was used as a negative control because it is not part of the immunogen formulation, but contains a His-tag. The production of MSP5 was referenced (line 178).

# R. Statistical analysis- Collapsing the data into a single data point for each parameter (PCV or PPE) for each animal results in a loss of data points. For example, PCV and PPE was determined daily for each animal, yet the statistical analysis was done with the maximum decrease in PCV for each animal or the maximum PPE for each animal, thus resulting in only one data point for each animal for each category and thus an overall loss of data and potentially statistical power. Admittedly this is unlikely to change the outcome of the analysis given the lack of differences between groups, but is still worth consideration.

**A.** We admit the loss of data in this analysis but we chose this methodology to present the results due to the absence of differences between groups.

R. Table 3: Please clarify maximum PPCV decrease. Is this the % drop in PCV? Standard deviation, as a measure of the amount of variability is more appropriate in this case than standard error.

**A.** We made the changes in the manuscript. PPCV is the % drop in PCV.

# Minor suggestions

# R. PCV is by definition a percent and is generally represented by PCV, not PPCV. If the measurement is percent drop in PCV, just say “percent drop in PCV”.

**A.** We made the change in the manuscript.

# R. Line 47,77- The use of the word, multiplied, is unusual in this context. Suggest, using either “produced” or “amplified” instead.

**A.** We made the change in the manuscript.

# R. Line 102- Add GenBank accession numbers as reference for the genes.

**A.** We added the access in the manuscript (lines 106 to 107).

# R. Line 157-Add a space between dodecyl and sulfate

**A.** We made the change in the manuscript.

# R. Fig. 1- Overall this figure needs improvement. Specifically, there needs to be more space between the coomasie and the western blot as the numbers for the ladder for the western blot overlap with the coomasie. Suggest improving figure 1 and removing the supplemental results.

**A.** We improved the figure.

# Reviewer #2: Dear Editor,

# Please find below the manuscript "A vaccine using *Anaplasma marginale* subdominant type IV secretion system recombinant proteins was not protective against a virulent challenge" PONE-D-19-26977, after complete correction.

# The authors developed two formulations containing *A. marginale* type IV secretion system (T4SS) subdominant proteins: VirB9.1, VirB9.2, VirB10, VirB11, and Ef-Tu T4SS beind expressed in *E. coli*.

# This proteins were absorbed on a Quil A® adjuvant (aqueous saponin adjuvant - G1) and and Montanide ISA 201 ready-to-use adjuvants for Water-in-Oil-in-Water emulsion (G2) and control groups Quil A® (G3) and ISA 201 (G4), respectively.

# Recent reports describe that conserved membrane proteins that are subdominant in *Anaplasma* species, such as VirB9 and VirB10, are constituents of the Type 4 secretion system (T4SS) that is conserved amongst many intracellular bacteria and performs essential functions for invasion and survival in host cells (Crosby et al 2018). Therefore, here this study is important.

# In addition, the work used free healthy cattle and also splentomized calves with parasitized erythrocytes from *A. marginale*, besides to explain the participation of constituents of the Type 4 secretion system (T4SS) on the immune response against *A. marginale* virulent.

# However I would like to request some explanations:

# R. line 52 -54 " Such protection was correlated with induction of high titers of IgG2 opsonizing antibodies against *A. marginale* surface epitopes and macrophage activation mediated by CD4+ T cells [4,11,12]". To compare the works (4,11,12) and the challenge test employed. Thus, it is important to insert on the discussion section.

**A.** The authors of reference 11 did not perform a challenge. They demonstrated that in *A. marginale* outer membrane-vaccinated cattle, VirB9, VirB10, and CTP are recognized by serum IgG2 and stimulate memory T-lymphocyte proliferation and gamma interferon secretion. Reference 12 is a review.

# R. line 94 - 100 and 241 -267 "In silico analysis of T4SS proteins sequences". I would suggest the authors to consider the inclusion of figure or a table with the sequences.

**A.** We included the sequences in the manuscript as supporting information (S1 Table).

# R. line 172 - 182 "Immunization...I would like that authors described about the adsorption methods of the vaccine formulations employed.

It´s necessary to describe on the material and methods section.

**A.** We described the methods in the manuscript (lines 187 to 191).

# R. Line 187 - 188 "Each challenge dose was adjusted to 107 parasitized erythrocytes in a 2 mL final volume and inoculated SC to the cattle, on day 105 after the first inoculation. Why? Others reports (Santos et al 2013; Crosby et al 2018) employed 1 x 10 5 for both dominant and subdominant epitopes. it is important to insert discussion section.

**A.** In the cited reports, the protection conferred by different antigens is evaluated in mice. In this species, the challenge dose and the challenge via are different than in cattle. Also in Crosby et al 2018, the mice are challenged with *A. phagocytophilum* that has tropism by a different cell (netrophils). The challenge dose depends on the species and via used. Different *A. marginale* challenge doses in cattle are evaluated in Abdala et al 1990.

# R. line 341-349 "Despite the correlation between IgG2 and protective immunity, both vaccine formulations evaluated in this work induced a strong IgG2 response but failed to mitigate the course of infection. Other subdominant proteins from the OM, AM854 and AM936, were able to induce an immune response comparable to that induced by the whole OM, based on high levels of IgG2; however, they also failed to protect against the challenge [37]" Here, I would like that authors described about the IgG2 production by dominant epitopes, and to compare with obtained data this work (subdominant epitopes).

**A.** We incorporated the description in the manuscript (lines 379 to 386).

# R. line 377 - 383 "Another cause of vaccine failure in this study may have been the inability of the immunogen to generate antibodies to block the parasite entry to the host cell. The most successful vaccines target highly conserved epitopes required by the pathogenic parasites for their host cell entry [42]". Here, I would like that authors described about the MSP-2 variants and the CD4(+)-T-cell epitopes that may be responsible for all or part of the A. centrale vaccine efficacy.

**A.** We incorporated the description in the manuscript (lines 419 to423).
