# Supplementary material for: A vaccine using Anaplasma marginale subdominant type IV secretion system recombinant proteins was not protective against a virulent challenge
Source: PLoS One. 2020 Feb 21;15(2):e0229301. doi: 10.1371/journal.pone.0229301 (PMC7034839; doi:10.1371/journal.pone.0229301)
Supplement: Attachment — Submitted filename: PONE D_19_26977R1_Reviewer#1.docx [file pone.0229301.s005.docx]

Dear Editor,

I'm satisfied with the authors responses and recommend the publication of the work. However, I suggest a minor revision as described in the attached. Grateful for the opportunity to evaluate this work.

Please find below the manuscript "A vaccine using Anaplasma marginale subdominant type IV secretion system recombinant proteins was not protective against a virulent challenge" PONE-D-19-26977R1, after complete correction.

Reviewer:

Line 52 – 54 Here, I would like that authors excluded "protection", because mentioned authors did not perform the challenge test. If possible, to replace the reference 12 by : Linkage between Anaplasma marginale Outer Membrane Proteins Enhances Immunogenicity but Is Not Required for Protection from Challenge. Clin Vaccine Immunol. 2013 May; 20(5): 651–656. Noh et al 2013.

Line 202-203 Noh et al 2013 (Linkage between Anaplasma marginale Outer Membrane Proteins Enhances Immunogenicity but Is Not Required for Protection from Challenge) it also describes about the challenge test of bovine (Holstein steers) and also can be used to discute the work
