# Supplementary material for: A vaccine using Anaplasma marginale subdominant type IV secretion system recombinant proteins was not protective against a virulent challenge
Source: PLoS One. 2020 Feb 21;15(2):e0229301. doi: 10.1371/journal.pone.0229301 (PMC7034839; doi:10.1371/journal.pone.0229301)
Supplement: Attachment — Submitted filename: Response to Reviewers.docx [file pone.0229301.s006.docx]

Reviewer #1

Line 15: Suggest replacing “multiplied” with “produced” or “amplified”.

We made the change in the manuscript.

Line 16: suggest replacing “vector” with “carry” or “transmit”

We made the change in the manuscript.

Line 18: Replace “works” with “work”

We made the change in the manuscript.

Line 23 and 26: Cattle from G1 and G2 were inoculated with a mixture of 50 ug of each recombinant protein with QuilA and Montanide adjuvants. Edit as follows: Cattle from G1 and G2 were inoculated with a mixture of 50 ug of each recombinant protein with QuilA or Montanide adjuvants, respectively.

We made the change in the manuscript.

Line 26: “Cattle received four doses…”. I suggest: Cattle received four immunizations…”

We made the change in the manuscript.

Line 28, line 245, 294, 298, 300, 311, 314, 319 and throughout the manuscript, for clarity, I suggest using immunization instead of inoculation when referring to administration of the antigens. This more specific language helps differentiate between immunization and challenge.

We made the changes in the manuscript.

Line 289: “(B) Western blot revealed with…” suggest “(B) Western blot using anti-His-tag….”

We made the change in the manuscript.

Line 312: Replace Babesia bovis with B. bovis

We made the change in the manuscript.

Line 315: Replace Anaplasma marginale with A. marginale

We made the change in the manuscript.

Reviewer #2

Line 52 – 54 Here, I would like that authors excluded "protection", because mentioned authors did not perform the challenge test. If possible, to replace the reference 12 by : Linkage between Anaplasma marginale Outer Membrane Proteins Enhances Immunogenicity but Is Not Required for Protection from Challenge. Clin Vaccine Immunol. 2013 May; 20(5): 651–656. Noh et al 2013

We replace the reference 12 and exclude the reference 11 because it has not challenge test. (line 55).

Line 202-203 Noh et al 2013 (Linkage between Anaplasma marginale Outer Membrane Proteins Enhances Immunogenicity but Is Not Required for Protection from Challenge) it also describes about the challenge test of bovine (Holstein steers) and also can be used to discute the work.

The challenge dose, the challenge via (subcutaneous or intravenous), the challenge method (infected blood or infected ticks) and the challenge time post-immunization are different in the bibliography. However, in all works *A. marginale* OM induced protection (Tebele et al 1991, Brown et al 1998, Ducken et al 2015, Albarrak et al 2012, Noh et al 2013). For this reason we decide not include the point in the discussion.
